# Post-COVID-19 EMU: Economic Distancing by Parallel Currencies

**DOI:** 10.1007/s10272-020-0939-4

**Published:** 2020-12-01

**Authors:** Thomas Mayer, Gunther Schnabl

**Affiliations:** 1 Flossbach von Storch Research Institute, Ottoplatz 1, 50679 Köln, Germany; 2 Institute for Economic Policy, University of Leipzig, Grimmaische Straße 12, 04109 Leipzig, Germany

## Abstract

The coronavirus crisis has caused new distress in the European Economic and Monetary Union (EMU), as the southern part of the EMU has been hit stronger than the northern part. The common currency prevents nominal exchange rate adjustment in response to the asymmetric shock. Policymakers have therefore taken recourse to large-scale financial transfers. Based on the lessons from the German monetary union, this article proposes instead the introduction of parallel currencies to facilitate relative price changes. Parallel currencies in the south would allow an increase in competitiveness of the south via real depreciation. The introduction of a parallel currency in Germany would lead to capital inflows and a real appreciation of the *new German mark.* The pre-EMU pressure for structural adjustments and productivity gains would be restored.

The coronavirus crisis has caused new distress in the European Economic and Monetary Union (EMU). Italy and other southern European countries accuse northern European countries, in particular Germany, of not doing enough to help in economic crises. The €750 billion Next Generation EU plan, which was announced as “a magnificent signal of solidarity and willingness to reform” (European Commission, 2020a) may bring temporary relief, but it will not address the main problem of the euro area: real economic divergence. This has been further aggravated by other coronavirus countermeasures, including border closures that have interrupted tourist flows to southern European countries for a prolonged and indefinite period of time.

In the short term, asymmetric shocks in a monetary union can be softened by targeted credit from the European Central Bank (ECB) or other unconventional monetary policy operations (Sonnenberg and Schnabl, 2019). Yet, in the longer run, the growing real divergence in the European monetary union cannot be countered by financial means. Transfers and cheap central bank credit (with softened collateral conditions) will facilitate the postponement of necessary reforms and the restructuring of enterprises (Acharya et al., 2012). Increasing real divergence is likely to increase dissatisfaction about the EMU in southern European countries and may induce voters to elect populist politicians promising a remedy through an EMU exit.

**Gunther Schnabl**, Leipzig University, Germany.

As a result, the EMU could break apart. However, a sudden break-up of the monetary union would cause major turbulences in financial markets and have far-reaching political consequences at the European and global level. In our view, it would be preferable to safeguard against such a cataclysmic event by introducing parallel currencies as a safety valve. Parallel currencies can increase the flexibility of the EMU where needed and allow for the release of pressure that could otherwise blow it up.

Parallel currencies are a widely observed phenomenon in developing countries (Agénor, 1990; Xaiyavong and Toyoda, 1990). The emergence of parallel currencies has been associated with the demand for foreign currency to purchase illegal imports (Boulding, 1947) and the strong rise of the domestic money stock relative to foreign money stock (Bleyer, 1978). More recently, the future of Bitcoin and Libra as parallel currencies has also been discussed (Mayer et al., 2019). Against this, we explore the potential for parallel currencies to introduce more flexibility into the EMU set-up with a view to softening real economic divergence. For this purpose, we build upon the experience of the German monetary union.

## Lessons from the German monetary union for the EMU

The Germans have their experience with a heterogenous monetary union (Sinn, 1992). On 18 May 1990, the West and East German finance ministers signed a treaty establishing the German Economic, Monetary and Social Union (GEMSU), which entered into force on 1 July 1990 and established the

Figure 1

**Primary income per capita in Bavaria and Saxony-Anhalt**

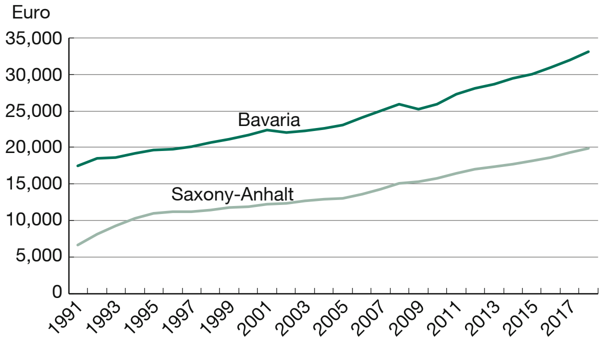

Source: Statistisches Bundesamt, 2020.

Figure 2

**Nominal GDP per capita of Germany, Italy and Greece**

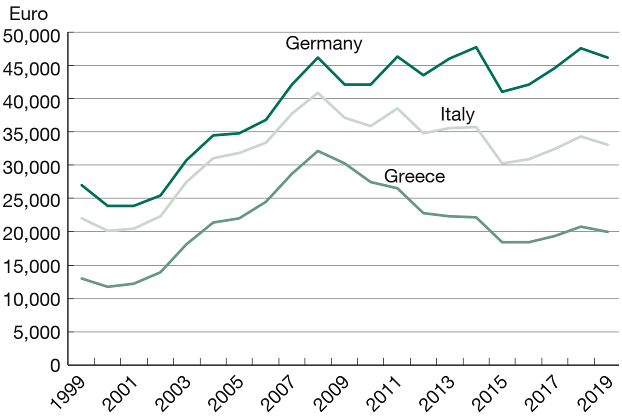

Source: Thompson Reuters Datastream, 2020

Figure 3

**Real gross domestic product of France, Germany, Greece, Italy and Spain**

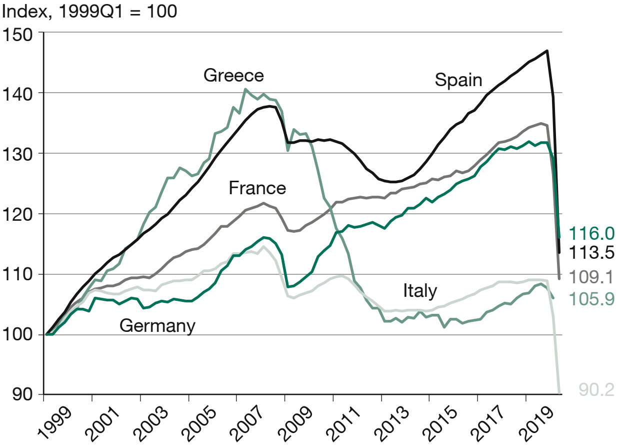

Source: Macrobond, 2020.

Deutsche Mark as official legal tender in the German Democratic Republic. The experience with GEMSU provides important insights for the future of the European Monetary Union.

In 1990, the East German economy was expected to adjust rapidly and to catch up quickly with its West German counterpart. Chancellor Helmut Kohl promised "blooming landscapes" within four years. This proved to be wishful thinking. Uncompetitive and unable to devalue its exchange rate, the East German economy tanked and unemployment spiked. Until today, productivity, income and wealth levels have not caught up by far. Tax revenues in Eastern Germany lag far behind the level of the Western part of the country (Schnabl and Sepp, 2020).

Figure 1 shows the primary income (before taxes and transfers) per capita for Bavaria, the federal state with the highest level in the Western part of Germany, and for Saxony-Anhalt, the state with the lowest level in the Eastern part of Germany. The per capita primary income gap was €10,928 in 1991. By 2018, it had widened to €13,239.

Only nine years after the GEMSU, eleven European countries with different economic structures entered the EMU, with rapid economic convergence again expected. As before, this proved to be wishful thinking. After a consumption boom in the southern EMU from 2001 to 2007, which for a while suggested economic convergence (Belke et al., 2005), the southern European economies fell behind. Among them, Italy and Greece in particular have been suffering from a persistent lack of competitiveness since 2008 (Schnabl, 2019). While income per capita of Italy and Greece were about €4,600 and €13,000, respectively, lower than in Germany in 1999, the gaps increased to roughly €13,000 and €20,000, respectively, by 2019 (see Figure 2).

Now, the pandemic further aggravates the problem. As the southern European economies rely more heavily on tourism and have a larger share of small and medium-sized enterprises than their northern neighbours, they are hit harder, with their competitiveness declining even further. While the German economy was 16% bigger in mid-2020 in real terms than at the beginning of 1999, when the EMU was launched, the Italian economy was almost 10% smaller. Thus, the gap in real GDP between the two countries has increased substantially since the beginning of the EMU (Figure 3). The divergence is likely to increase further in the future. The European Commission's (2020b) gloomy 2020 growth forecasts are substantially brighter for Germany (-6.3%) than for Italy (-11.2%), Spain (-10.9%), Greece (-9.0%) and Portugal (-9.8%).

When instead of blooming landscapes an economic desert emerged in eastern Germany, the government applied huge volumes of fertilizer. To prop up the economy and people's incomes, they transferred approximately €1.5 billion of taxpayer money over the course of 23 years after unification to the East. This amounted to more than 12 times the GDP of the Eastern German economy in 1991. Nevertheless, unemployment in Eastern Germany kept rising, reaching a peak of 20.6% in 2005. Until today, 1.5 million mostly young and educated people, equivalent to 10% of the total population, have left the territory of the former German Democratic Republic. This has eventually reduced unemployment, but it has left the economy in Eastern Germany emaciated.

Similarly, since the euro crisis, southern European economies were propped up with large credits from newly created euro rescue funds and have received continuous funding support from the ECB via extensive asset purchases and long-term credit financing with softened requirements for the quality of collateral. But they have failed to grow. Many young people have left southern Europe and will continue to do so. In response to the coronavirus crisis, European politicians have designed a new €750 billion economic recovery package. But as the experience of eastern Germany over the last 30 years and that of southern Europe over the last decade shows, this will most likely fail to induce growth, create employment or save the euro.

### Relative price changes needed

Income support alone will not restore economic growth when economic structures are not adapted to new circumstances. What is required in the face of asymmetric shocks are relative price changes, either via nominal exchange rate adjustments or adjustments of wages and prices (Mundell, 1961). For the EMU to persist, the southern euro area economies need a reduction of their relative prices, which would require wage repression in the south and/or wage increases in north.[1]

Since the introduction of the euro, the relative prices of the southern euro area countries (except France) have increased against Germany instead of decreased (Figure 4). After the outbreak of the European financial and debt crisis, this process was – despite hardships as in the case of Greece – only partially reversed. As a result, the southern euro area countries suffered from a loss of competitiveness. This will most likely be aggravated by the fall-out from the coronavirus crisis, which leaves countries with large service sectors and many small companies at a disadvantage to countries with larger manufacturing and information and communications technology sectors and bigger companies.

1 Kenen (1969; 2003) suggested that a common fiscal policy could compensate for the imperfect fit of a one-size-fits-all monetary policy in a currency union as far as stabilisation policy is concerned. But historical experience from Germany and Italy suggests that fiscal policy cannot compensate for lasting structural differences.

Figure 4
**Relative prices of France, Italy, Portugal, Spain and Greece versus Germany**

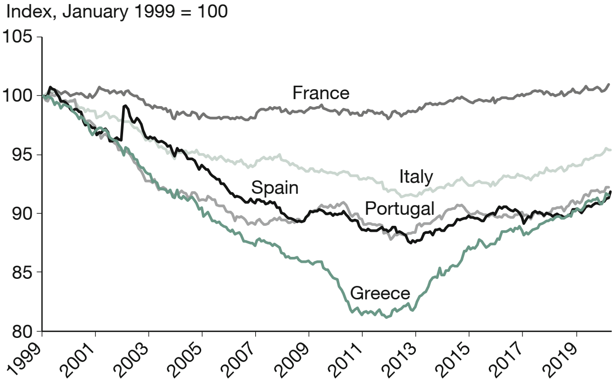

Note: Falling lines indicate prices increasing relative to Germany.

Source: IMF and down calculations based on consumer prices.

The resulting external deficits could be easily financed with large debt capital inflows or credit provided by the ECB. Even more credit and transfers in the wake of the coronavirus crisis may indeed soften the financial pain in the short run, but they will only postpone the necessary real economic adjustment and further increase the need for it. Dissolution of the EMU would quickly adjust relative prices by depreciating the southern European currencies. But an outright exit of the southern European countries from the EMU would cause substantial turmoil in financial markets and political crises. Hence, unconventional measures to avoid the breakup of the euro are needed.

### Parallel currencies in the south

The relative prices could be reduced by launching parallel currencies in the southern euro area countries, which could take the form of non-redeemable government bonds without interest and in small denominations. In Italy, such instruments have been proposed and dubbed 'mini-BOTs' (derived from *Buoni del Tresoro*, the name for Italian government bonds; Mayer, 2019a). When the government funds part of its expenditures by issuing these mini-BOTs and accepts them as payment for a part of tax liabilities – based on the share they have in taxpayers' incomes – these instruments could become a parallel currency for domestic use. Mini-BOTs could be issued electronically, like other government bonds (*Buoni del Tesoro Poliennale* or BTPs) or they could be issued as paper money.

Since the parallel currency would most likely be deemed a less attractive store of value, the mini-BOTs would depreciate against the euro, inducing a decline in euro prices and a reduction of Italy's relative prices vis-à-vis its euro area trading partners. To follow this argument, assume that the government pays a part of social transfers and salaries in mini-BOTs. As sellers of goods and services to these groups would now receive part of their sales revenue in mini-BOTs as well, they would also pay part of the wages to their employees and of the costs of their inputs in this currency. Profit margins for recipients of euros from sales abroad could increase when costs for domestic inputs have declined, but many of them would probably opt for cutting their euro prices to raise their market share. Thus, a decline of the prices of Italian goods relative to those of its euro area trading partners would follow.

As long as there is slack in the economy, inflation of goods priced in the parallel currency for domestic use would remain low, allowing the nominal depreciation of the mini-BOTs to induce a real depreciation and to exert downward pressure on goods priced in euro for exports. Foreign demand for Italian goods would rise, in particular for services in the tourism sector, which constitutes a substantial share of economic activity in the Italian economy.

While economic growth and employment would benefit, the Italian government would receive less taxes in euros and may no longer be able to service its euro-denominated debt. Hence, the introduction of the parallel currency would need to go along with debt reduction. Restructuring would be painful. But a policy of debt reduction along the lines of the 1933 Chicago Plan with turning the euro into a digital central bank currency could achieve the same effect at much lower costs.[2]

A large part of euro area government debt could be taken out of the market by placing it permanently on the balance sheet of the ECB as a cover pool for existing paper money and a new central bank digital currency. Following the conversion of existing bank money into central bank money through purchases of outstanding government bonds by the ECB, the initial central bank money stock could be increased over time according to a fixed rule. Both the creation of the digital euro money stock and the rule for its increase could be enshrined in a smart contract embodied in the digital euro (Mayer, 2019b).

## Parallel currencies in the north

Alternatively, the German Bundesbank could begin to withdraw from the ECB's asset purchasing programmes. A ruling of the German Federal Constitutional Court (2020) on 5 May 2020 opened the path to gradual withdrawal. The Court found good reasons for a breach of mandate by the ECB's purchases of public sector bonds, and it gave the ECB three months to explain itself. Meanwhile the ECB explanation given to the German government and parliament was regarded as sufficient by both, but a new complaint is now before the German Constitutional Court.

2 See also Benes and Kumhof (2012).

The withdrawal of the Bundesbank from these programmes could lead to capital flight to Germany in anticipation of a break-up of the euro, which is likely to come along with a depreciation of southern and an appreciation of northern European currencies. To fend off a bank run from southern Europe into Germany, the Bundesbank would have to close the existing TARGET2 interbank payment system and accept euro payments from other member countries only if backed by gold or foreign exchange as collateral (Sinn and Wollmershäuer, 2012). If southern European central banks lack the collateral needed to make transfers, euro payments could no longer be made from southern Europe to Germany.

TARGET2 eliminates the restrictions set in the Exchange Rate Mechanism (ERM) of the European Monetary System (1979-1998), which preceded the EMU (Ungerer et al., 1990; Gros and Thygesen, 1998). In the ERM, currencies were tied together with fixed exchange rates, around which they could fluctuate within a limited band. Credit facilities[3] were strictly limited in size and time so that countries experiencing balance of payment deficits were eventually forced to devalue their currencies against the Deutsche Mark, which served as an anchor for the other currencies. The adjustment of the central parities was explicitly foreseen. TARGET2 now allows the unlimited funding of balance of payments deficits, presently at zero costs, since the interest rate on liabilities within the system is set at the marginal refinancing rate of the ECB, which is zero (Sinn and Wollmershäuser, 2012). With the outbreak of the current coronavirus crisis, the imbalances within the TARGET2 payment system have started to grow again strongly.

Balance of payment imbalances have already increased substantially since the euro crisis of 2010-12. Now, Italy's liabilities within the TARGET payment system have reached €536 billion (end of June 2020) and Germany's claims €1019 billion (end of July 2020). If Germany ties the funding of balance of payments imbalances in the future to collateral, which southern European countries do not have, payments can only continue via the foreign exchange market, where exchange rates are allowed to adjust freely. To this end (and to avoid the 'dollarisation' of European trade payments), Germany could introduce a new, freely floating currency. Let us call it the *new mark*. If Germany would remain in the EMU and use the new mark as a parallel currency, a financially disruptive and politically divisive breakup of the euro area could be avoided. Payments in euros could still be made between southern Europe and Germany.

3 The very short-term credit facility provided an unlimited method to support foreign exchange intervention. Repayment was due 45 days after the end of the month of intervention. Prolongation was possible for another three months. The short-term monetary support (14 billion ECU) was available in case of temporary balance of payment deficits. The medium-term financial assistance (11 billion ECU) was to assist in case of balance of payments deficits only if the stability of the common market was at risk.

If inflows would match outflows, there would be no additional demand for the new mark. The exchange rate between the euro and the new mark would remain unchanged. But if payment flows from southern Europe to Germany were larger than from Germany to southern Europe, the difference would have to be made by exchanging euros against new marks. The new mark would appreciate against the euro.[4]

Given persistent appreciation expectations, the new mark would probably replace the euro as a store of value in Germany, but payments within Germany and abroad could still be made in euros. The appreciation of the new mark would boost German demand for foreign products priced in euros, the competitiveness of southern Europe would increase and private payment flows from Germany to southern Europe would be generated. Southern European countries would have an incentive to increase the competitiveness of their economies to avoid too large of a loss of income. Thus, the pressure for structural adjustment, which existed before the EMU but was reduced by the ability to cheaply finance internal and external deficits, would be restored. If southern euro area countries managed to run balance of payments surpluses, they could even eliminate the need for new marks and allow Germany to run down its big TARGET2 surpluses.

As before, the entire group of EMU member countries could reduce their large government debt by placing part of their government bonds permanently on the ECB's balance sheet as a 100% cover for the euro. They could establish safeguards for a further monetisation of debt in order to support confidence in the euro.

## Outlook

If policymakers continue on their present course, more funds will be necessary to slow a further decline in incomes of southern European countries. This would put additional financial burdens on tax payers in the northern European countries, against which voters will probably rebel when economic recovery from the present recession remains sluggish or the next recession hits.

Despite more transfers, southern European economies most likely will shrink further, as a fundamental restructuring of government expenditure and of enterprises will continue to be postponed. This will further strengthen populist politicians and may eventually give them a mandate to take these countries out of the monetary union. The euro and, possibly, also the European Union, could be destroyed.

4 A system of matching payments among European countries existed in 1950-1958 in the form of the so-called European Payments Union (Albers et al., 1980). When payment imbalances exceeded certain thresholds and the embedded credit system reached its limits, they had to be made in US dollars or in gold.

To avert this, politicians will need to think outside the box. Parallel currencies would provide an appropriate approach to adjust long-lasting differences in competitiveness within the EMU. It would restore competitive pressure through exchange rate appreciation in the north, which before the euro introduction incentivised northern European enterprises to increase productivity and allow countries with lower productivity growth to support employment through exchange rate depreciation.
